# Guiding Inspiratory Flow: Development of the In-Check DIAL G16, a Tool for Improving Inhaler Technique

**DOI:** 10.1155/2017/1495867

**Published:** 2017-11-16

**Authors:** Mark Jeremy Sanders

**Affiliations:** Clement Clarke International Limited, Edinburgh Way, Harlow, Essex CM20 2TT, UK

## Abstract

Portable inhalers are divisible into those that deliver medication by patient triggering (pMDIs: a gentle slow inhalation) and those that use the patient's inspiratory effort as the force for deaggregation and delivery (DPIs: a stronger deeper inspiratory effort). Patient confusion and poor technique are commonplace. The use of training tools has become standard practice, and unique amongst these is an inspiratory flow meter (In-Check) which is able to simulate the resistance characteristics of different inhalers and, thereby, guide the patient to the correct effort. In-Check's origins lie in the 1960s peak* expiratory *flow meters, the development of the Mini-Wright peak flow meter, and inspiratory flow assessment via the nose during the 1970s–1980s. The current device (In-Check DIAL G16) is the third iteration of the original 1998 training tool, with detailed and ongoing assessments of all common inhaler resistances (including combination and breath-actuated inhaler types) summarised into resistance ranges that are preset within the device. The device works by interpolating one of six ranges with the inspiratory effort. Use of the tool has been shown to be contributory to significant improvements in asthma care and control, and it is being advocated for assessment and training in irreversible lung disease.

## 1. Introduction

Portable (i.e., pocket-able) inhalers can be divided into those that deliver the medication by patient triggering (e.g., pressurised metered dose devices, pMDIs) and those that use the patient's own inspiratory effort as a force for deaggregation and delivery (e.g., dry powder inhaler devices, DPIs). Each of these two groups requires a different inspiratory effort: pMDIs require a gentle slow inhalation and DPIs require a stronger deeper effort [1]. Patients are known to confuse the techniques when prescribed both types of devices [2].

The importance of inspiratory flow as an influencer of successful inhaler technique is highlighted in the European Respiratory Society/International Society for Aerosols in Medicine (ERS/ISAM) task force document [3]. The closing summary sentences read (this author's emphases): “*healthcare providers should ensure that their patients *can and will* use these devices correctly. This requires that the clinician: is aware of the devices that are currently available to deliver the prescribed drugs; *knows the various techniques* that are appropriate for each device; *is able to evaluate* the patient's inhalation technique to be sure they are using the devices properly; and *ensures that the inhalation method* is appropriate for each patient*.”

The ERS/ISAM recommendations task the clinician with a series of demanding actions beyond diagnosis and prescription. One of the main consequences, however, of failing to achieve these is poor inhaler technique that delivers not the correct dose of medication but poor asthma control [1]. Such is the complexity and variety of modern inhaler therapies that train-the-trainer workshops and the use of specially designed training tools have become commonplace. The device tools available include placebo inhalers, replica inhalers with inspiratory flow whistles, flow whistle inhaler add-ons, simulators, electronic airflow detectors, and—uniquely—an inspiratory flow meter which is able to simulate the resistance characteristics of different inhalers.

The importance of inhaler technique training is highlighted in the Global Initiative for Asthma guidance [4] and in national asthma guidance [5–8]. Training is usually provided by medical or pharmacy staff but is generally provided in a punctuated fashion with the need for frequent retraining [9]. Some training tools permit ongoing use outside the clinic and facilitate greater training continuity [10]. In addition to inhaler competency training it is important that patient education covers adherence but, allowing for the retraining element, consultation time available for education may be compromised. It is important that competency assessment and training tools align with educational messages.

This article focuses on the history and development of the unique clinic/pharmacy based inspiratory flow meter training device (In-Check DIAL G16, Clement Clarke International Limited).

## 2. In-Check DIAL G16

**Figure 1:**
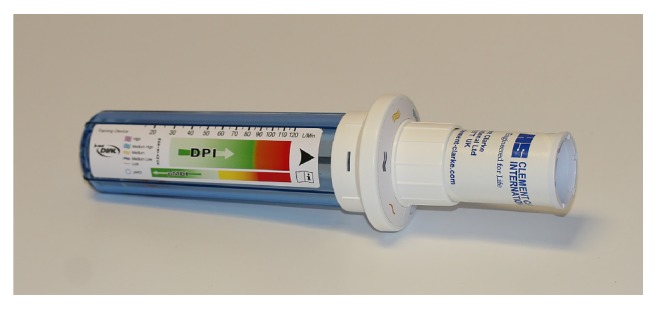
*In-Check DIAL G16 (Clement Clarke International Limited).* In-Check DIAL G16 is a multipatient clinic/pharmacy device using disposable, single-patient mouthpieces and is used for assessing the inspiratory effort of a patient inhaling through a selected inhaler. The DIAL can be set to resemble the resistance of the inhaler, and the appropriate inhalation—slow and gentle for pMDI and fast and strong for DPI—can be coached. See online animation: https://www.youtube.com/watch?v=bGCfCGw9 h24.

The In-Check DIAL G16 device is a specialised inspiratory peak flow meter (Figure 1). In many ways it resembles the functioning of a regular peak expiratory flow (PEF) meter. The inspiratory device, however, originates from work in the 1960s [11] using an adaptation of the original large, heavy Wright peak (expiratory) flow meter to determine peak inspiratory flow (PIF) and was used to assess response to bronchodilator therapy in chronic airways obstruction. The now-familiar tubular, “miniature” version was commercialised in the late 1970s following a decade of developmental research [12]. This work led to a special version of the “Mini-Wright” being developed for peak nasal inspiratory flow measurement and named after the allergist Youlten [13]. A similar adaptation was used by Depledge [14] to investigate the utility of a PEF/PIF ratio in assessing bronchodilator effectiveness.

A Clement Clarke product improvement programme in the 1990s produced the basic In-Check mechanism: a coaching tool to train patients to make an inspiratory flow effort consistent with the requirements of their specific inhaler device(s) [15]. This device was refined in the late 1990s to create the In-Check DIAL [16, 17], and has now evolved—particularly as a result of the availability of today's novel inhalers—to its present form as the In-Check DIAL G16 [18], with a measurement range of 15−120 L/min (±10 L/min). A further, newer development is the In-Check M, developed from the In-Check DIAL, specific to pMDI use and simulating this inhaler format only.

Throughout the development of the In-Check DIAL products, Clement Clarke amassed a large database of resistance profiles of different inhalers. The basic methodology of resistance profiling was initially described by Clark and Hollingworth [19] and that methodology has been widely adopted by industry when describing device resistance. By conducting all of the measurements on the same calibrated equipment under identical conditions, a relative comparison and classification of inhaler devices by their resistance profile can be assembled. While there are international standards for peak expiratory flow meters [20] there are none for peak inspiratory flow measurement. Clement Clarke, as the sole manufacturer of inspiratory flow meters, has developed the* de facto* standard.

Early versions of In-Check DIAL identified optimal flow rates for the individually represented inhalers by the inclusion of a restrictive adapter in the dial but a series of developments shifted the emphasis toward the* clinically effective range*. Optimal flow rates are mainly justified on* in vitro* delivery data that do not always reflect the dose response behaviour of the drug* in vivo* [21]. In addition, such was the increase in the number and type of available inhaler devices [22]; there was a clear need to group together inhalers by ranges of device resistance. A summary of these development changes is given in Table 1.

Many inhaler devices are now available with a number of formulation choices, and each of the formulations can have subtle differences in delivery characteristics [30]. Similarly, combination inhaler use is increasing and in some instances the optimal delivery condition for one component may be different to that of another component [31]. Likewise, breath-actuated and flow rate triggered devices may have two flow rate characteristics: the first being the effort required to actuate the triggering mechanism and the second the flow rate required to aerosolise and deliver the drug(s) effectively. Breath-actuation flow rate is device-engineered, and trigger rates tend to be set early in the inspiration and relatively low (e.g., pMDI Easi-Breathe 20 L/min, pMDI Autohaler 30 L/min, and DPI Nexthaler 35 L/min [32]): the implication being that, for deaggregation and aerosolisation of dry powders, the effectiveness of the inspiratory manoeuvre overall may be related to a subsequent higher flow rate and/or acceleration of flow [32–34].

The next stage of product development became, therefore, one of determining how many ranges and the definition of the ranges. Clement Clarke approached this through a consultative process with academic and industry members and the consideration of published reference sources (Table 2).

**Figure 2:**
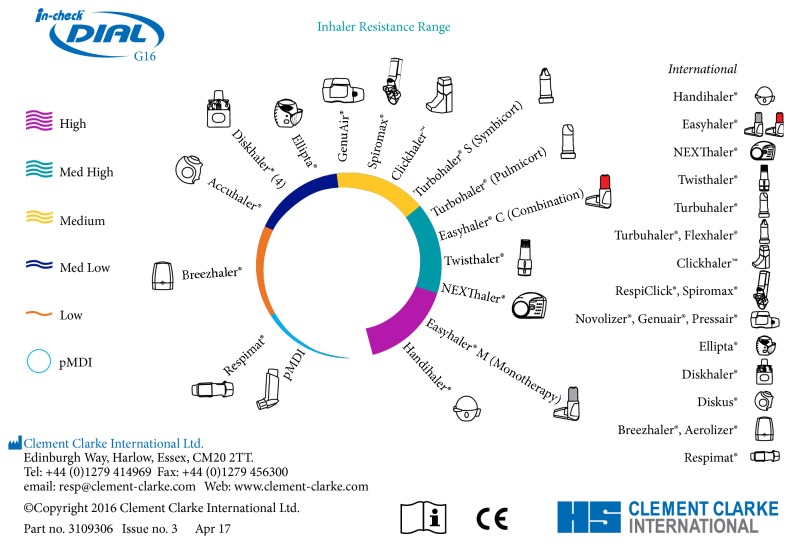
In-Check DIAL G16 Information Card.

Various resistance classifications of DPIs have been proposed, including simple 3-point (low, medium, and high) [35] and 4-point definitions (low, medium, medium/high, and high) [3]. The mathematical determinations conducted by Clement Clarke revealed, however, that a broader schema of a single representation of pMDIs, including the specialised soft mist inhaler Respimat [36] and five representations of DPI device resistance (low, medium-low, medium, medium-high, and high), was appropriate (Figure 2). The output of the calculations was compared with published data and despite the determination of published values under frequently noncomparable experimental conditions, there was a good degree of overall conformity. A confounding element is that certain devices have resistance variants (e.g., Turbuhaler M2 and M3) [29]: in some countries and for some of the product range, the original Turbuhaler (often identified as M2) has been replaced with the new Turbuhaler (identified as M3 or the trade name Flexhaler). The newer device has an improved design, permitting actuation quantities of 200 *μ*g budesonide and 6 and 12 *μ*g formoterol to be replaced by equivalents of 180, 4.5, and 9 *μ*g, respectively, based on the amount of medication that actually leaves the mouthpiece. Also, when generic formulations are made available in existing inhaler platforms, engineering changes are introduced to ensure comparability with the brand-originator (e.g., to the Easyhaler DPI delivering either a single or combination formulation). This will require great clarity for those referencing inhaler resistances in the future.

**Figure 3:**
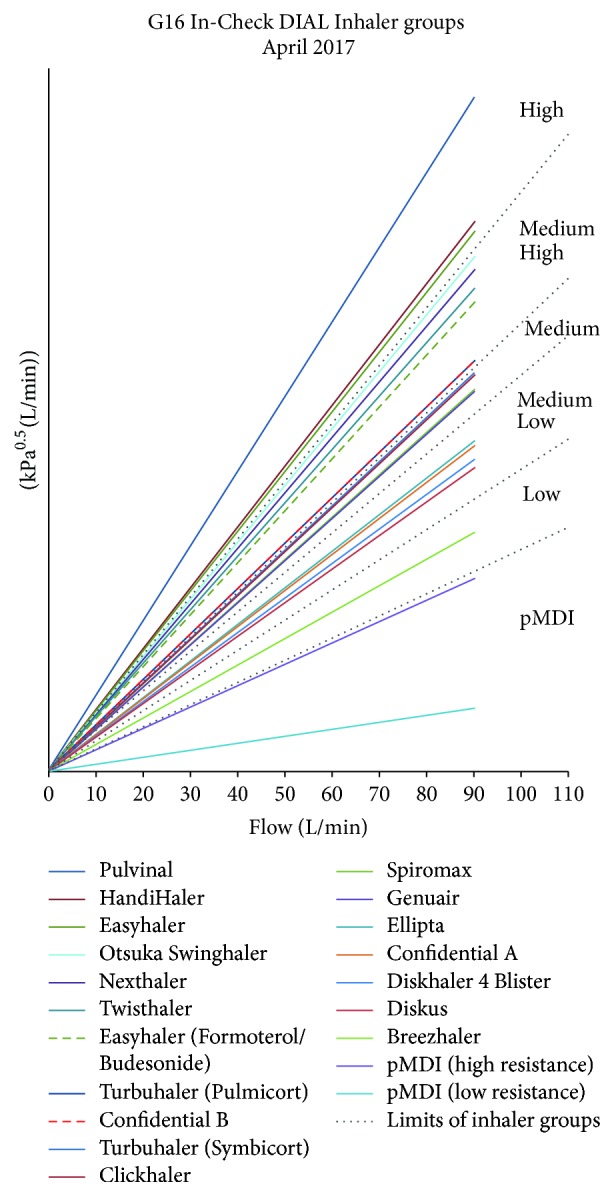
In-Check DIAL G16 ranges of device resistance.

The In-Check DIAL G16 resistance profile data are therefore based on fully comparable in-house measurements made under standard conditions by Clement Clarke scientists. For combination inhalers the G16 provides the flow rate ranges which, from published clinical experience, demonstrate efficacy and for breath-actuated devices, to trigger their function. Detailed resistance profile data and relative representations are shared with device originators (Figure 3).

## 3. Discussion

In-Check DIAL has been widely used in asthma clinics since the early 2000s. The data from the Isle of Wight study [10, 37, 38] demonstrate the value of institutional and home-use training aids: pharmacists were initially instructed on how to use the inhalers themselves and then trained to measure a patient's ability to use the prescribed inhaler using the In-Check DIAL. The entirety of the project delivered a reduction of >50% in emergency admissions and a fall in asthma-associated deaths of 75%, alongside reduced short-acting bronchodilator use. This research provided remarkable, clinically quantifiable improvements in patient well-being. The daily use scenarios to which the In-Check DIAL can be put (Table 3) are directly supportive to this type of medicines use review [37].

In-Check DIAL can also be used in the assessment of treatment options for chronic obstructive pulmonary disease (COPD) patients [18]. COPD may be characterised during exacerbations by a suboptimal peak inspiratory flow [22] that would affect a patient's ability to receive medication effectively and has been shown to be independently predictive of hospital COPD readmissions [39]. New research with the G16 is being directed to coaching COPD patients to improve their peak inspiratory flow and to determine if its measurement can help personalise inhaler selection [18].

## 4. Conclusion

Training that can be delivered swiftly, establishing good inhaler technique alongside understanding, may free-up time during consultation sessions to address adherence explanations. The ultimate aim is to establish individual asthma management plans built upon good inhaler competency and good adherence.

## Figures and Tables

**Table 1:** Timeframe and scope of In-Check DIAL development.

	In-Check DIAL model (date)		
	5 T&S (Q4 1999)	6 T&S (Q3 2010)	G16 (Q3 2016)
Body	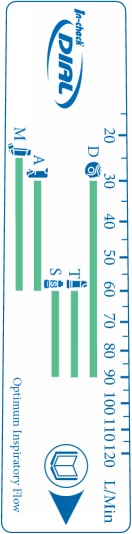	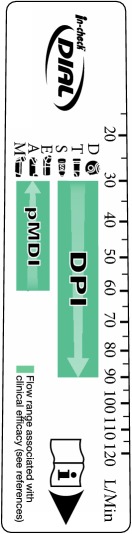	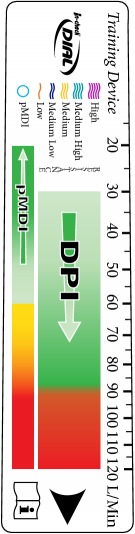

Dial	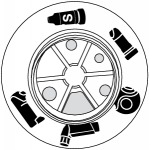	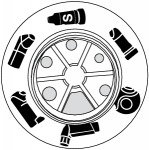	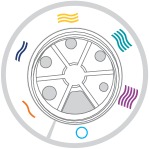

Basis	Optimum flow rate	Clinically effective range	

Number and Inhalers covered	5	6	16
	Accuhaler (Diskus) Autohaler pMDI Turbuhaler (M2) Turbuhaler (M3) and conventional pMDIs	Accuhaler (Diskus) Autohaler pMDI Easi-Breathe pMDI Turbuhaler (M2) Turbuhaler (M3) and conventional pMDIs	15 inhaler devices and conventional pMDIs (see Figures 2 and 3)

**Table 2:** Example third party dry powder inhaler resistance measures (kPa^1/2^/L·min^−1^).

DPI device	Inhaler resistance measures (kPa^1/2^/L·min^−1^) [Ref. number]						
	[23]	[24]^*∗*^	[25]	[26]	[27]	[28]^¶^	[29]
Breezhaler (Neohaler)	0.02	0.0197				0.019	0.017
Clickhaler				0.0394			
Diskhaler					0.032		
Diskus (Accuhaler)		0.0275		0.0249	0.034	0.026	0.027
Ellipta		0.0275					0.027
Easyhaler				0.0424		0.037	0.050
Genuair (Pressair) [Novoliser]	0.031				[0.028]	0.029	0.031
HandiHaler		0.0510				0.05	0.058
Nexthaler	0.036				0.042	0.033	0.036
Spiromax (Respiclick)			0.0313				
Turbuhaler Pulmicort		0.0382		0.0337	0.043	0.039	0.039
Turbuhaler Symbicort (Flexhaler)			0.0355			0.033	0.035
Twisthaler							0.044

*Note.* Resistances determined by one source may differ from another (*∗* converted from cm H_2_O^1/2^/L·min^−1^, ¶ determined from Figure  2 in [28]).

**Table 3:** Use scenarios and maintenance of the In-Check DIAL G16.

In-Check DIAL G16	
Use scenarios	In-Check maintenance and components
To comply with Guidelines that require inhaler technique training to be conducted To provide objective inhaler technique/flow rate data To assess whether poorly-controlled patients have adequate inhaler technique To conduct training in the absence of specific trainer/demonstrator devices To guide/tailor inhaler device selection To support trainer-related reimbursement	*See Instructions for Use for details*: (i) *Wash* in warm mild detergent solution for a maximum of 5 minutes. Agitate the water (ii) *Rinse* in warm water and shake to remove any excess water by holding at the end furthest away from the DIAL (iii) *Allow to dry* thoroughly before reuse *Additional components*: (i) Disposable mouthpieces (ii) Filtered mouthpieces (iii) Restriction adaptors for specific DPI resistance requirements
